# Association between lung function and the risk of atrial fibrillation in a nationwide population cohort study

**DOI:** 10.1038/s41598-022-07534-4

**Published:** 2022-03-07

**Authors:** Su Nam Lee, Seung-Hyun Ko, Sung-Ho Her, Kyungdo Han, Donggyu Moon, Sung Kyoung Kim, Ki-Dong Yoo, Yu-Bae Ahn

**Affiliations:** 1 Division of Cardiology, Department of Internal Medicine, St. Vincent’s Hospital, The Catholic University of Korea, 93, Jungbu-daero, Paldal-gu, Suwon, Gyunggi-do 16247 Republic of Korea; 2 Division of Endocrinology and Metabolism, Department of Internal Medicine, St. Vincent’s Hospital, The Catholic University of Korea, 93, Jungbu-daero, Paldal-gu, Suwon, Gyunggi-do 16247 Republic of Korea; 3 Department of Statistics and Actuarial Science, Soongsil University, Seoul, Republic of Korea; 4 Division of Pulmonary and Critical Care Medicine, Department of Internal Medicine, St. Vincent’s Hospital, The Catholic University of Korea, Seoul, Republic of Korea

**Keywords:** Health care, Medical research

## Abstract

We investigated the association between lung function and atrial fibrillation (AF) in 21,349 adults without AF aged ≥ 40 years who underwent spirometry. The study participants were enrolled from the Korean National Health and Nutritional Examination Survey between 2008 and 2016. The primary outcome was new-onset non-valvular AF identified from the National Health Insurance Service database. During the median follow-up of 6.5 years, 2.15% of participants developed new-onset AF. The incidence rate of AF per 1000 person-years was inversely related to the forced expiratory volume in 1 s (FEV_1_), forced vital capacity (FVC), and FEV_1_/FVC quartile. After adjustment for multiple variables, the AF risk in the lowest FEV_1_ quartile was 1.64-fold higher than that in the highest quartile (hazard ratio (HR) 1.64 (95% confidence interval (CI) 1.26–2.12) for lowest FEV_1_ quartile). The lowest quartile of FVC had 1.56-fold higher AF risk than the highest quartile (HR 1.56 (95% CI 1.18–2.08) for lowest FVC quartile). Although the lowest FEV_1_/FVC quartile was associated with an increased risk of AF in the unadjusted model, this increased risk was not statistically significant in the multivariable analysis. Compared to those with normal lung function, participants with restrictive or obstructive lung function had 1.49 and 1.42-fold higher AF risks, respectively. In this large nationwide cohort study, both obstructive and restrictive patterns of reduced lung function were significantly associated with increased AF risk.

## Introduction

Atrial fibrillation (AF) is the most common arrhythmia in adults worldwide^1^. AF is associated with increased risks of stroke, heart failure, and death, leading to a high economic burden^2^. AF occurs in an estimated 2–4% of adults^1^. And the incidence rate is expected to gradually increase with the increasing age of the population. Therefore, undiagnosed AF should be recognized in the general population to prevent AF-related complications^3^. The risk of AF is increased by several factors, including age, male sex, obesity, smoking, alcohol consumption, hypertension, diabetes mellitus (DM), chronic kidney disease (CKD), coronary artery disease, valvular heart disease, heart failure, obstructive sleep apnea, and chronic obstructive pulmonary disease (COPD)^2^.

AF frequently occurs in patients with airway diseases, such as COPD and asthma. Patients with asthma and COPD have approximately 1.5- and twofold higher AF risks than those without asthma and COPD, respectively^4,5^. Several factors, such as smoking, hypoxia, inflammation, and increased use of beta-2 agonists, contribute to the development of AF in patients with airway diseases^6^. Reduced lung function, i.e., low forced expiratory volume in 1 s (FEV_1_) and forced vital capacity (FVC), is associated with heart failure and ischemic heart disease (IHD), which are risk factors for AF. In addition, reduced lung function is associated with stroke, which is the most common complication of AF^7–9^. Previous studies have reported that lung function is inversely related to AF risk^10–12^. However, these studies focused on FEV_1_ and FVC instead of lung function categories, and were conducted in Western populations.

We analyzed a nationwide population-based cohort to investigate the association between reduced lung function and the AF risk in an adult population using two linked Korean National Health Databases: Korean National Health and Nutrition Examination Survey (KNHANES) and National Health Insurance Service (NHIS). To the best of our knowledge, this is the first study to investigate the association between restrictive or obstructive lung function impairment and AF in a nationwide population-based cohort with detailed anthropometric and laboratory results.

## Results

### Baseline characteristics

Table 1 presents the baseline characteristics of the 21,349 participants. Among the study population, 10.2% and 13.1% had restrictive and obstructive lung function impairments, respectively. Compared to those with normal lung function, participants with restrictive or obstructive lung function impairment had higher prevalence of old age, male sex, current smoking, heavy alcohol consumption, low household income, unemployment, low education, DM, hypertension, dyslipidemia, asthma, prior stroke and prior IHD. Obesity and abdominal obesity were significantly more common in participants with restrictive lung function impairment than the other groups.

**Table 1:** Baseline characteristics of the study population.

Variable	Normal (*n* = 16,365)	Restrictive (*n* = 2178)	Obstructive (*n* = 2806)	*p*-value
Age ≥ 65 years	3479 (21.3)	873 (40.1)	1652 (58.9)	< .0001^a,b,c^
Male	6294 (38.5)	1031 (47.3)	2058 (73.3)	< .0001^a,b,c^
**Smoking**				< .0001^a,b,c^
Nonsmoker	10,734 (65.6)	1280 (58.8)	889 (31.7)	
Ex-smoker	2978 (18.2)	497 (22.8)	1078 (38.4)	
Current smoker	2653 (16.2)	401 (18.4)	839 (29.9)	
**Alcohol consumption**				< .0001^a,b,c^
None	4768 (29.1)	793 (36.4)	898 (32.0)	
Mild to moderate	10,342 (63.2)	1208 (55.5)	1607 (57.3)	
Heavy	1255 (7.7)	177 (8.1)	301 (10.7)	
Moderate physical activity	6417 (39.2)	796 (36.6)	1208 (43.1)	< .0001^a,b,c^
Household income (lowest quartile)	2713 (16.6)	540 (24.8)	888 (31.7)	< .0001^a,b,c^
Occupation, yes	10,590 (64.7)	1214 (55.7)	1596 (56.9)	< .0001^a,b^
High education	9693 (59.2)	1037 (47.6)	1166 (41.6)	< .0001^a,b,c^
Diabetes mellitus	1768 (10.8)	503 (23.1)	520 (18.5)	< .0001^a,b,c^
Hypertension	5712 (34.9)	1130 (51.9)	1379 (49.1)	< .0001^a,b,c^
Dyslipidemia	3221 (19.7)	521 (23.9)	532 (19.0)	< .0001^a,c^
Asthma	311 (1.9)	90 (4.1)	246 (8.8)	< .0001^a,b,c^
Prior stroke	219 (1.3)	68 (3.1)	74 (2.6)	< .0001^a,b^
Prior ischemic heart disease	338 (2.1)	106 (4.9)	118 (4.2)	< .0001^a,b^
**Anthropometric measurements**				
Obesity	5920 (36.2)	1150 (52.8)	837 (29.8)	< .0001^a,b,c^
Abdominal obesity	6841 (41.8)	1302 (59.8)	991 (35.3)	< .0001^a,b,c^
Height (cm)	155.9 ± 9.1	155.7 ± 9.0	158.7 ± 8.9	< .0001^b,c^
Weight (kg)	57.7 ± 10.6	60.6 ± 11.4	58.6 ± 10.4	< .0001^a,b,c^
Body mass index (kg/m^2^)	24.1 ± 2.9	25.3 ± 3.2	23.7 ± 2.7	< .0001^a,b,c^
Waist circumference (cm)	77.5 ± 9.0	81.9 ± 9.6	79.7 ± 8.8	< .0001^a,b,c^
SBP (mmHg)	120.8 ± 16.4	126.7 ± 17.3	125.6 ± 16.7	< .0001^a,b^
DBP (mmHg)	77.7 ± 10.2	78.0 ± 10.4	76.1 ± 10.5	< .0001^b,c^
**Laboratory findings**				
Fasting blood glucose (mg/dL)	100.2 ± 22.4	108.2 ± 30.3	103.6 ± 22.8	< .0001^a,b,c^
Serum creatinine (mg/dL)	0.8 ± 0.2	0.9 ± 0.3	0.9 ± 0.4	< .0001^a,b,c^
eGFR (mL/min/1.73m^2^)	90.4 ± 16.6	87.7 ± 18.3	85.8 ± 17.0	< .0001^a,b,c^
White blood cell (× 10^9^/L)	6.0 ± 1.7	6.4 ± 1.8	6.5 ± 1.8	< .0001^a,b^
Hemoglobin (g/dL)	13.9 ± 1.5	14.0 ± 1.6	14.4 ± 1.4	< .0001^a,b,c^
Total cholesterol (mg/dL)	195.9 ± 35.9	193.0 ± 37.2	188.8 ± 36.4	< .0001^a,b,c^
HDL cholesterol (mg/dL)	49.5 ± 11.8	46.5 ± 11.1	47.0 ± 11.6	< .0001^a,b^
Triglyceride (mg/dL)	118.7 (117.6–119.7)	132.8 (129.8–136.0)	126.0 (123.5–128.7)	< .0001^a,b,c^
ALT (IU/L)	19.0 (18.9–19.2)	21.4 (21.0–21.9)	19.4 (19.0–19.7)	< .0001^a,c^
AST (IU/L)	21.5 (21.4–21.6)	23.1 (22.7–23.4)	22.7 (22.4–23.0)	< .0001^a,b^

Values are presented as *n* (%) or mean ± standard deviation. SBP, systolic blood pressure; DBP, diastolic blood pressure; eGFR, estimated glomerular filtration rate; HDL, high density lipoprotein; AST, aspartate transaminase; ALT, alanine transaminase. ^a^Significant for Normal vs Restrictive, ^b^significant for Normal vs Obstructive, ^c^significant for Restrictive vs Obstructive.

Among the study population, 2.15% had new-onset AF during a median follow-up of 6.5 years (interquartile range = 4.5–8.5 years) (Table 2). Compared to those without AF, participants with new-onset AF were more likely to exhibit old age, male sex, current smoking, heavy alcohol consumption, unemployment, obesity, and low income and education levels. Participants with AF had significantly worse baseline lung function and were more likely to have DM, hypertension, asthma, stroke, CKD, and IHD than those without AF.

**Table 2:** Baseline characteristics according to the development of AF.

Variable	No AF (*n* = 20,890)	AF (*n* = 459)	*p*-Value
Age ≥ 65 years	5746 (27.51)	258 (56.21)	< .0001
Male	9147 (43.79)	236 (51.42)	0.0011
**Smoking**			0.0003
Nonsmoker	12,654 (60.57)	249 (54.25)	
Ex-smoker	4420 (21.16)	133 (28.98)	
Current smoker	3816 (18.27)	77 (16.78)	
**Alcohol consumption**			< .0001
None	6280 (30.06)	179 (39.00)	
Mild to moderate	12,929 (61.89)	228 (49.67)	
Heavy	1681 (8.05)	52 (11.33)	
Moderate physical activity	8227 (39.38)	194 (42.27)	0.2112
Household income (lowest quartile)	3978 (19.04)	163 (35.51)	< .0001
Occupation, yes	13,160 (63.00)	240 (52.29)	< .0001
High education	11,728 (56.14)	168 (36.60)	< .0001
Diabetes mellitus	2708 (12.96)	83 (18.08)	0.0013
Hypertension	7948 (38.05)	273 (59.48)	< .0001
Dyslipidemia	4191 (20.06)	83 (18.08)	0.2945
Asthma	618 (2.96)	29 (6.32)	< .0001
Prior stroke	344 (1.65)	17 (3.70)	0.0007
Prior ischemic heart disease	529 (2.53)	33 (7.19)	< .0001
**Lung function**			< .0001
Normal	16,105 (77.09)	260 (56.64)	
Restrictive	2096 (10.03)	82 (17.86)	
Obstructive	2689 (12.87)	117 (25.49)	
**Anthropometric measurements**			
Obesity	7712 (36.9)	195 (42.3)	0.0146
Abdominal obesity	8887 (42.5)	247 (53.8)	< .0001
Height (cm)	156.28 ± 9.09	156.27 ± 9.99	0.9986
Weight (kg)	58.10 ± 10.68	59.48 ± 10.66	0.0061
Body mass index (kg/m^2^)	24.19 ± 2.91	24.67 ± 3.05	0.0004
Waist circumference (cm)	78.21 ± 9.10	81.07 ± 9.37	< .0001
SBP (mmHg)	121.85 ± 16.63	128.25 ± 17.98	< .0001
DBP (mmHg)	77.49 ± 10.25	77.49 ± 10.92	0.9964
**Laboratory findings**			
Fasting blood glucose (mg/dL)	101.47 ± 23.55	102.12 ± 20.02	0.5616
Serum creatinine (mg/dL)	0.84 ± 0.24	0.89 ± 0.35	< .0001
eGFR (mL/min/1.73 m^2^)	89.58 ± 16.84	85.37 ± 19.14	< .0001
White blood cell (× 10^9^/L)	6.07 ± 1.70	6.04 ± 1.66	0.7651
Hemoglobin (g/dL)	14.00 ± 1.53	14.05 ± 1.62	0.4956
Total cholesterol (mg/dL)	194.75 ± 36.14	189.93 ± 36.20	0.0047
HDL cholesterol (mg/dL)	48.90 ± 11.77	46.89 ± 11.80	0.0003
Triglyceride (mg/dL)	120.91 (119.97–121.86)	124.99 (118.61–131.71)	0.2239
ALT (IU/L)	19.31 (19.18–19.44)	19.12 (18.31–19.97)	0.6678
AST (IU/L)	21.81 (21.71–21.90)	23.00 (22.30–23.71)	0.0006

Values are presented as *n* (%) or mean ± standard deviation. AF, atrial fibrillation; SBP, systolic blood pressure; DBP, diastolic blood pressure; eGFR, estimated glomerular filtration rate; HDL, high density lipoprotein; AST, aspartate transaminase; ALT, alanine transaminase.

### Lung function quartile and AF

**Figure 1:**
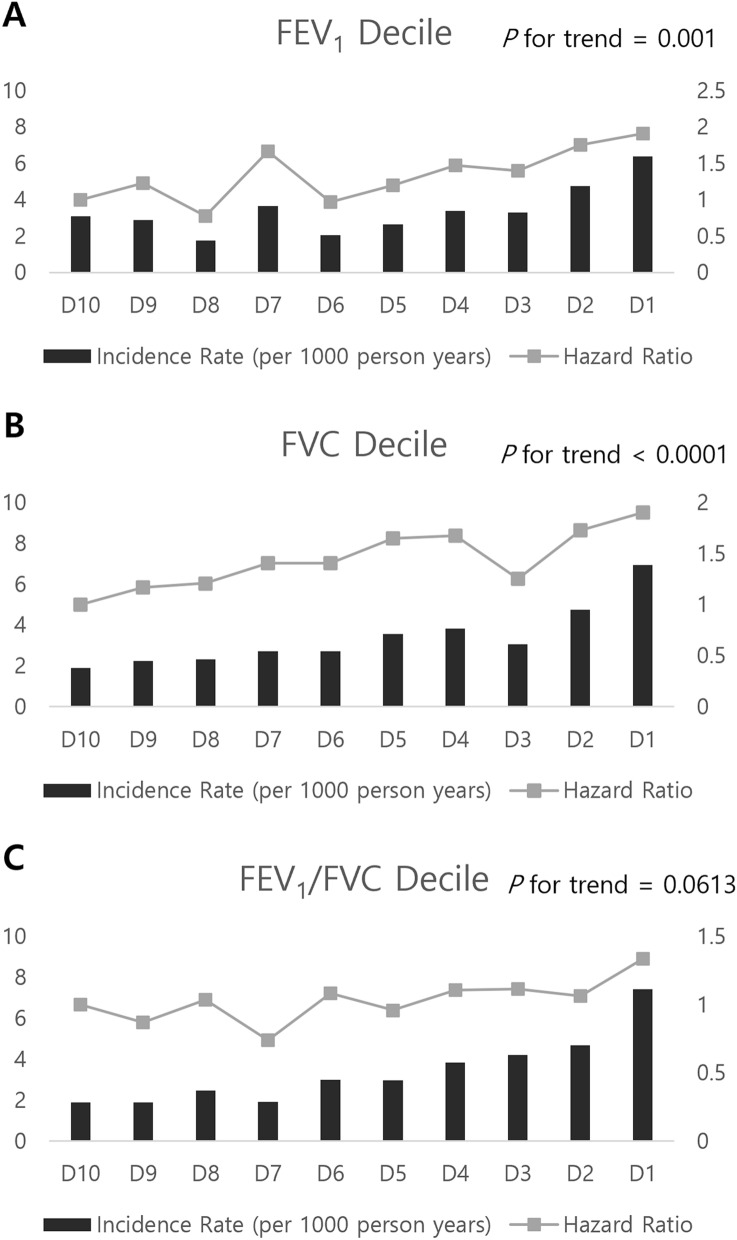
Incidence rate and hazard ratio of atrial fibrillation according to the decile of lung function measurements. (**A**) FEV_1_, (**B**) FVC, and (**C**) FEV_1_/FVC. All HRs adjusted for covariates including age, sex, BMI, household income, education, exercise, alcohol consumption, smoking, hypertension, DM, dyslipidemia, white blood cell count, eGFR, stroke, IHD, and asthma.

The incidence of AF was inversely related to FEV_1_, FVC, and FEV_1_/FVC quartiles. From the highest to lowest quartiles, the incidence rates were 2.82, 2.55, 3.10, and 5.08 for FEV_1_; 2.11, 2.64, 3.52, and 5.32 for FVC; 1.87, 2.60, 3.51, and 5.71 for FEV_1_/FVC (Table 3). Therefore, lung function quartiles were inversely associated with AF risk. After adjustment for multiple variables, the AF risks were 1.64-fold higher for the lowest compared to the highest FEV_1_ quartile (HR 1.64 (95% CI 1.26–2.12) for the lowest FEV_1_ quartile), and 1.56-fold higher for the lowest compared to the highest FVC quartile (HR 1.56 (95% CI 1.18–2.08) for the lowest FVC quartile), respectively. Although the lowest FEV_1_/FVC quartile was associated with increased AF risk in the unadjusted model, this inverse association was not statistically significant in the multivariable analysis. Figure 1 shows the AF incidence rates and HRs by lung function deciles.

**Table 3:** Incidence rate and hazard ratios for the risk of atrial fibrillation according to quartiles of FEV_1_, FVC, and FEV_1_/FVC ratio.

	Number of case	Number of AF	IR of AF (per 1000 person years)	Hazard ratio (95% CI)			
				Unadjusted	Model 1	Model 2	Model 3
**FEV1**							
Q4 (highest)	5337	97	2.82	1	1	1	1
Q3	5337	87	2.55	0.91 (0.68–1.21)	1.13 (0.85–1.52)	1.13 (0.85–1.52)	1.11 (0.83–1.49)
Q2	5339	105	3.10	1.10 (0.83–1.45)	1.32 (1.00–1.74)	1.30 (0.98–1.72)	1.28 (0.96–1.69)
Q1 (lowest)	5336	170	5.08	1.80 (1.41–2.32)	1.71 (1.33–2.21)	1.70 (1.32–2.19)	1.64 (1.26–2.12)
**FVC**							
Q4 (highest)	5337	73	2.11	1	1	1	1
Q3	5338	90	2.64	1.25 (0.92–1.70)	1.27 (0.93–1.73)	1.25 (0.92–1.71)	1.24 (0.91–1.68)
Q2	5338	119	3.52	1.67 (1.25–2.24)	1.53 (1.14–2.05)	1.44 (1.07–1.94)	1.41 (1.05–1.90)
Q1 (lowest)	5336	177	5.32	2.53 (1.93–3.32)	1.75 (1.33–2.31)	1.60 (1.21–2.13)	1.56 (1.18–2.08)
**FEV1/FVC**							
Q4 (highest)	5337	66	1.87	1	1	1	1
Q3	5338	89	2.60	1.40 (1.02–1.92)	1.09 (0.79–1.51)	1.1 (0.80–1.52)	1.10 (0.80–1.51)
Q2	5350	119	3.51	1.89 (1.40–2.56)	1.14 (0.84–1.56)	1.16 (0.85–1.59)	1.15 (0.84–1.57)
Q1 (lowest)	5324	185	5.71	3.11 (2.35–4.12)	1.31 (0.96–1.79)	1.37 (1.00–1.88)	1.32 (0.96–1.82)

Model 1: Adjust by age, sex.

Model 2: Adjust by age, sex, body mass index, household income, education, alcohol consumption, smoking, diabetes mellitus, hypertension, dyslipidemia.

Model 3: Adjust by age, sex, body mass index, household income, education, alcohol consumption, smoking, diabetes mellitus, hypertension, dyslipidemia, white blood cell, estimated glomerular filtration rate, prior stroke, prior ischemic heart disease, asthma.

FEV_1_, forced expiratory volume in 1 s; FVC, forced vital capacity; AF, atrial fibrillation; IR, incidence rate (events/1000 person years); CI, confidence interval.

### Restrictive or obstructive lung function impairment and AF

**Figure 2:**
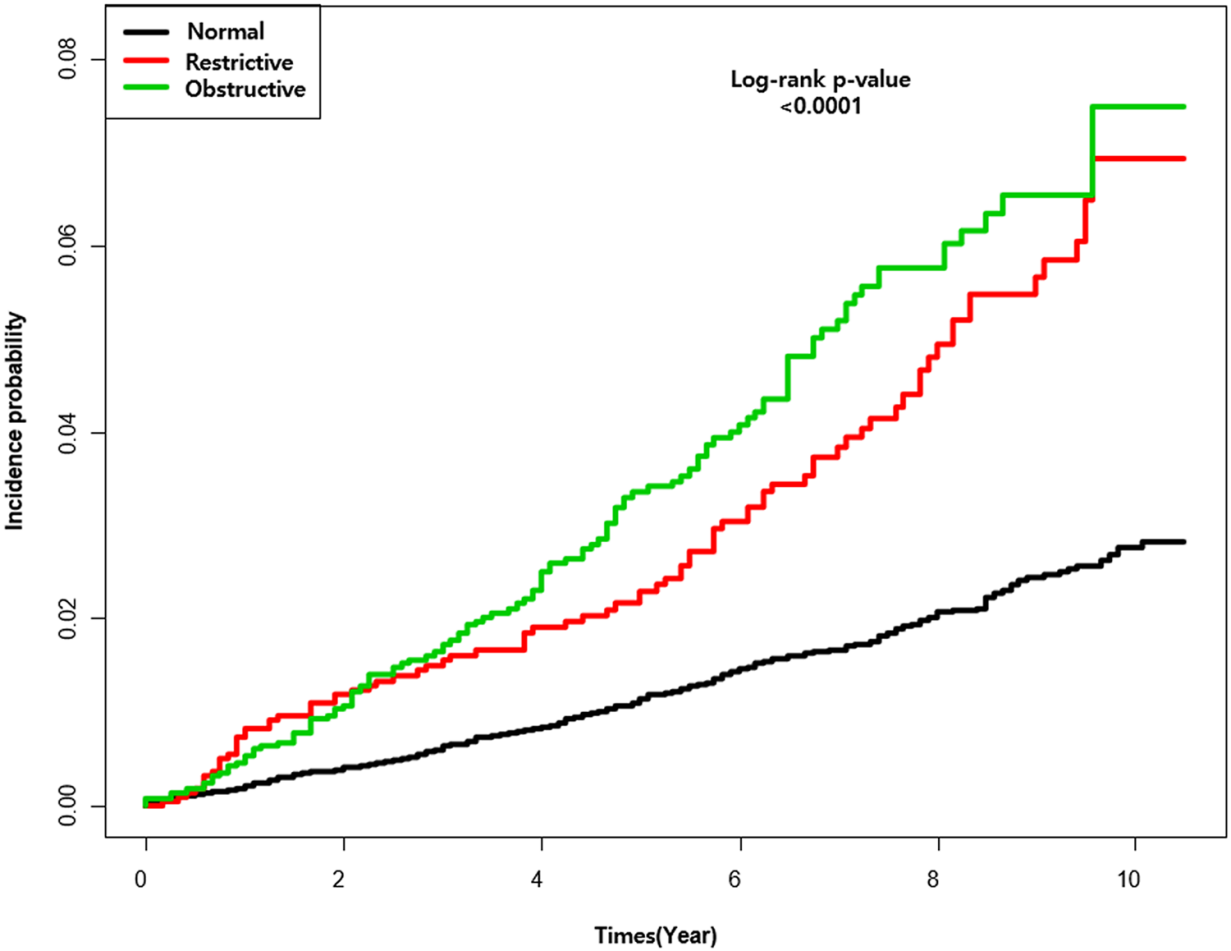
Clinical outcomes according to lung function.

Among participants with restrictive and obstructive lung function impairment, the incidence rates of newly diagnosed AF were 5.92 and 6.96 per 1000 person-years, respectively (Table 4). The age- and sex-adjusted HR for AF according to lung function impairment was 1.64 (95% CI 1.27–2.11) for restrictive lung function impairment and 1.42 (95% CI 1.11–1.81) for obstructive lung function impairment. After adjusting for age, sex, BMI, household income, education, exercise, alcohol consumption, smoking, hypertension, DM, dyslipidemia, stroke, IHD, asthma, white blood cell and eGFR, AF was significantly associated with reduced lung function (HR 1.49 (95% CI 1.15–1.92) for restrictive lung function impairment, HR 1.42 (95% CI 1.11–1.82) for obstructive lung function impairment, respectively)) (Table 4, Fig. 2).

**Table 4:** Incidence rate and hazard ratios for the risk of atrial fibrillation according to restrictive or obstructive lung function impairment.

	Number of case	Number of AF	IR of AF (per 1000 person years)	Hazard ratio (95% CI)			
				Unadjusted	Model 1	Model 2	Model 3
Normal	16,365	260	2.47	1	1	1	1
Restrictive or obstructive	4984	199	6.49	2.64 (2.16–3.18)	1.51 (1.24–1.85)	1.48 (1.21–1.82)	1.45 (1.18–1.78)
Normal	16,365	260	2.47	1	1	1	1
Restrictive	2178	82	5.92	2.39 (1.86–3.06)	1.64 (1.27–2.11)	1.52 (1.17–1.96)	1.49 (1.15–1.92)
Obstructive	2806	117	6.96	2.86 (2.30–3.55)	1.42 (1.11–1.81)	1.46 (1.14–1.86)	1.42 (1.11–1.82)

Model 1: Adjust by age, sex.

Model 2: Adjust by age, sex, body mass index, household income, education, alcohol consumption, smoking, diabetes mellitus, hypertension, dyslipidemia.

Model 3: Adjust by age, sex, body mass index, household income, education, alcohol consumption, smoking, diabetes mellitus, hypertension, dyslipidemia, white blood cell, estimated glomerular filtration rate, prior stroke, prior ischemic heart disease, asthma.

AF, atrial fibrillation; IR, incidence rate (events/1000 person years); CI, confidence interval.

### Subgroup analysis

**Figure 3:**
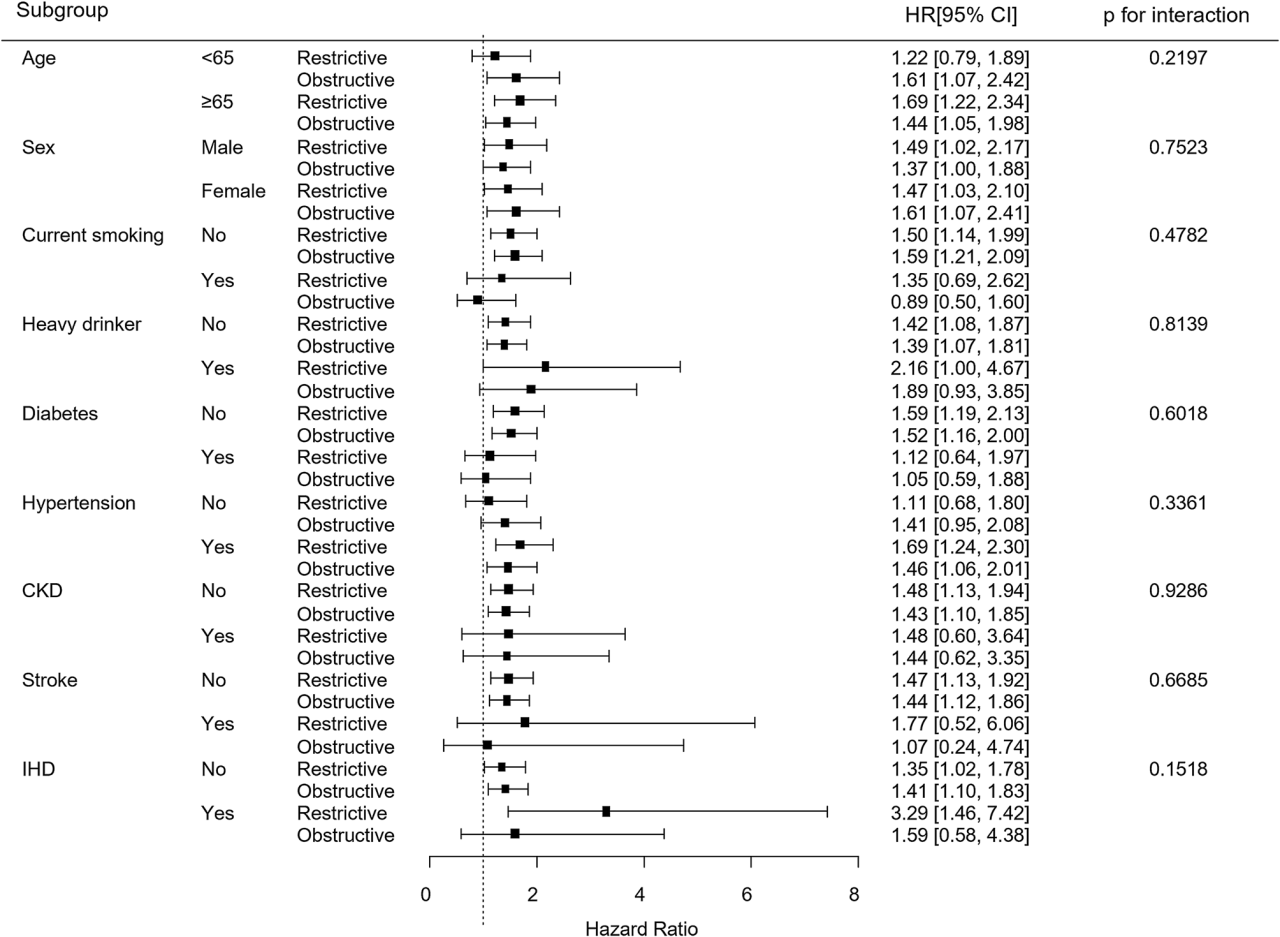
Subgroup analysis for atrial fibrillation risk. All HRs adjusted for covariates including age, sex, BMI, household income, education, exercise, alcohol consumption, smoking, hypertension, DM, dyslipidemia, white blood cell count, eGFR, stroke, IHD, and asthma. CKD chronic kidney disease, IHD ischemic heart disease.

Figure 3 shows the results of the subgroup analysis of reduced lung function and AF risk according to age, sex, smoking, alcohol consumption, DM, hypertension, CKD, stroke, and IHD. The association between reduced lung function and AF risk did not vary according to any of these factors. There were no significant interactions observed in any subgroup.

## Discussion

In this large national cohort study, we found that reduced lung function was associated with increased AF risk, with similar risks for obstructive and restrictive lung function impairments. Lung function quartiles were inversely related to the AF risk. The main finding of the present study was that individuals with obstructive or restrictive lung function impairments had approximately 1.4-fold higher AF risk than those with normal lung function.

Most studies of pulmonary function and AF risk have been multicenter cohort studies that mainly focused on the FEV_1_ and FVC values, rather than categories of lung function impairment^11–14^. Moreover, previous studies had significant differences in the FEV_1_ and FVC categories, follow-up duration and study design. A large Swedish population-based cohort study with a long follow-up duration of 24.8 years showed an inverse relationship between lung function and AF incidence by gender, but many patients were treated in an outpatient rather than an inpatient setting, hospitalized patients were excluded from the study^10^. The Atherosclerosis Risk In Communities (ARIC) study also reported an inverse relationship between lung function and AF, with a relatively high rate of AF development (11%) during the follow-up of 11 years. However, in the ARIC study, 73.8% of participants were White, who have a higher AF risk than Asians^12,13^. In addition, the ARIC investigators reported that obstructive lung function impairment was associated with increased AF incidence. However, they did not analyze restrictive lung function impairment in the study. In the Multi-Ethnic Study of Atherosclerosis (MESA) study, the AF incidence varied by race and was significantly lower in non-White patients compared to White patients^15^. In a cohort study conducted in specific regions of Korea, new-onset AF was identified in 1.7% of the population, and individuals in the lowest FEV_1_ quartile had 1.59-fold higher AF risk than those in the highest quartile, which is in line with the results of our study. However, in the previous study, AF was not defined using the ICD-10 code^14^. In the present nationwide population cohort study, including data from the baseline physical examination, laboratory findings, outpatient visits, hospitalization, and medication claims, the AF risk was significantly inversely associated with FEV_1_ and FVC quartiles after multivariable adjustment. Interestingly, patients with obstructive or restrictive lung function impairment had a 1.4-fold higher AF incidence than those with normal lung function.

The mechanisms underlying the association between decreased lung function and AF are unclear. However, previous studies have suggested several mechanisms, including hypoxia, systemic inflammation, increased sympathetic nerve activation, and the use of COPD treatments, such as beta-2 agonists and oral steroids^6^. Hypoxia is common in individuals with decreased lung function and causes pulmonary vasoconstriction, which leads to pulmonary hypertension and increased afterload to the right side of the heart^16,17^. Moreover, chronic hypoxia regulates the expression of hypoxia-inducible factors 1 and 2, the production of reactive oxygen species, blood pressure, and vascular inflammation^18^. Atrial structural remodeling plays a key role in the development of AF in patients with impaired lung function. In addition, elevated white blood cell count and C-reactive protein levels are associated with increased AF incidence^19^. Fogarty et al.^20^ reported that the C-reactive protein level is inversely related to lung function. The present study also showed that the white blood cell count was elevated in individuals with restrictive and obstructive lung function impairments, in line with previous studies. Sympathetic nerve activation, which is related to AF progression, is often found in patients with reduced lung function^21^. Inhaled beta-2 agonists and anticholinergics, the main treatments for COPD, are highly associated with tachyarrhythmia^22^. In addition, oral corticosteroids and theophylline increase the risk of AF^23^.

The present study of nationwide population showed increased AF incidence in patients with reduced lung function. Therefore, individuals with reduced lung function may be potential candidates for more meticulous screening of AF. To the best of our knowledge, this was the first study to investigate the association between obstructive or restrictive lung function impairment and AF risk using detailed anthropometric and laboratory results in a nationwide cohort.

This study has several limitations. First, clinical outcomes relied only on claims data and there might be missed diagnoses of asymptomatic and paroxysmal AF. Second, the pulmonary function tests used in this study could not assess the severity of COPD because of the pre-bronchodilator results. Third, this study was conducted with a single Asian ethnic group. Fourth, we measured lung function using only one method, spirometry without chest computed tomography, which indicates the presence or absence of bronchiectasis or emphysema. However, spirometry is a simple and easy method to check lung function, and trained examiners measured lung function with a predefined protocol. Fifth, medications that could increase the incidence of AF, such as beta-2 agonists and oral steroids were not considered. Sixth, compared to population without lung function impairment, those with obstructive or restrictive lung function impairment may have higher detection rate of AF, due to this group tends to get more medical attention and examination. Last, in this study, more subjects had minor reduced lung function than severely reduced lung function, because KNHANES data were obtained from subjects participating in a national survey. However, the KNHANES data can represent the public health of the entire population of Korea, which has significant implications in many respects^24^.

## Conclusions

We found that reduced lung function was an independent risk factor for AF in a nationwide population cohort study. Subjects with impaired lung function have a high risk of AF, and both obstructive and restrictive lung function impairment have similar AF risks. Future prospective research is needed to clarify the mechanism of association between reduced lung function and AF.

## Methods

### Source of the database and study population

We derived the data by cross-referencing KNHANES and NHIS. In this study, we used KNHANES data to collect study population and NHIS data to determine clinical outcomes. The KNHANES has been performed by the Korean Centers for Disease Control and Prevention at 3-year intervals since 1998 to monitor the general health and nutritional status of the civilian, noninstitutionalized Korean population^25^. Sampling units were households selected via a stratified, multistage probability-sampling design that considered geographic area, sex, and age group, by reference to household registries. NHIS is a social insurance payment system that covers approximately 97% of the Korean population. The Korean NHIS data include all national health checkup data and claim data, including drug prescriptions, diagnostic codes for the International Classification of Disease-10 (ICD-10) disease coding system, and claimed treatment details^26^. All KNHANES participants provided written informed consent for participation.

**Figure 4:**
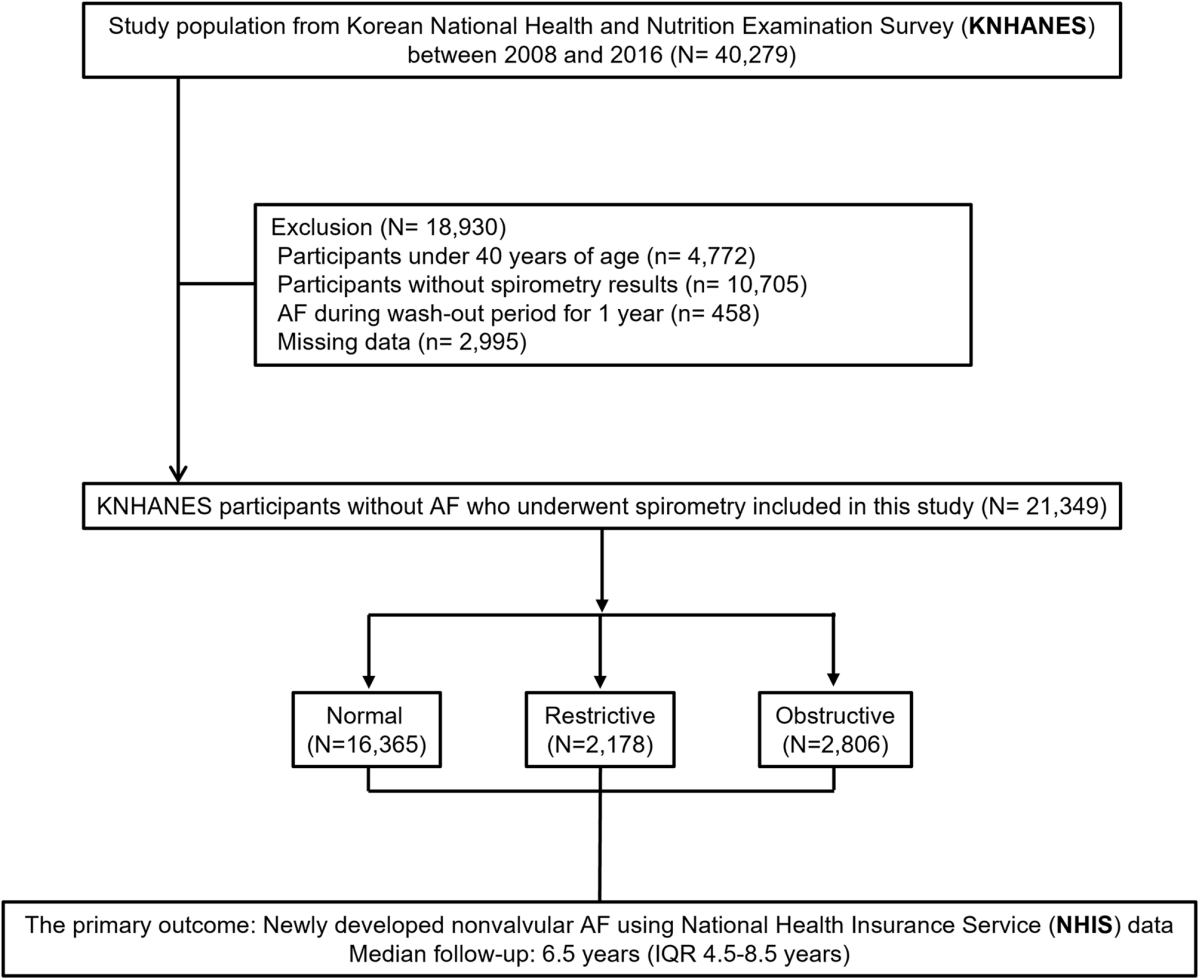
Sample recruitment from the database.

In this study, data from the KNHANES between 2008 and 2016 were used. We excluded participants among the 40,279 sample individuals as follow, aged < 40 years (*n* = 4772), participants without spirometry results (*n* = 10,705), participants who were already diagnosed with AF during wash-out period for 1 year (*n* = 458), or missing data (*n* = 2995) (Fig. 4).

The institutional review board of the Catholic University of Korea (IRB no: VC21ZISI0041) approved this study. The study was conducted in compliance with the Declaration of Helsinki. Informed consent was obtained from all participants at the time of survey collection.

### Clinical and laboratory measurements

Details of the KNHANES regarding health surveys, standardized physical examinations, laboratory tests, and definitions of risk factors have been described in a previous paper^25^. Specially trained examiners performed the physical examination by a standardized method. Body mass index (BMI) was calculated as participant body weight in kilograms divided by the square of height in meters. Obesity was defined as subjects with BMI more than 25 kg/m^2^^27^. Waist circumference (WC) was measured at the midpoint between the lowest rib and the anterior iliac crest in the standing position. Abdominal obesity was defined as subjects with WC more than 90 cm in men and more than 80 cm in women. The definition of abdominal obesity was a WC ≥ 90 cm in men and ≥ 85 cm^28^. However, our data were provided by masking in 10 cm increments, and the standard of 80 cm in women has been applied. The health-related behavior surveys included well-established questionnaires to determine the demographic and socioeconomic characteristics of the population. Smoking status was divided into three categories: nonsmoker, ex-smoker, or current smoker. Alcohol consumption was assessed based on the average number of alcoholic beverages and frequency of drinking. Heavy drinkers were defined as subjects who drank more than 30 g/day, and subjects drinking less than 30 g/day were classified as mild to moderate drinkers^29^. Moderate physical activity was defined as walking at least 150 min per week^29^. Household income was divided into quartile groups: lowest, lower middle, higher middle, and highest. Occupation status was divided into yes or no. High education level was defined as subjects who finished high school education or more.

DM was defined as a fasting glucose level ≥ 126 mg/dL, current use of antidiabetic medications, or a self-reported physician diagnosis of DM^30^. Hypertension was defined as systolic blood pressure ≥ 140 mmHg or diastolic blood pressure ≥ 90 mmHg, the current use of antihypertensive medications, or a self-reported physician diagnosis of hypertension^31^. Dyslipidemia was defined as total cholesterol ≥ 240 mg/dL, use of cholesterol-lowering medications, or a self-reported physician diagnosis of dyslipidemia. Asthma, prior stroke, and prior IHD were defined as a self-reported diagnosis by a physician in the health interview surveys^31^.

After overnight fasting, blood samples were collected from participants’ antecubital veins. The estimated glomerular filtration rate (eGFR) was calculated using the Modification of Renal Diet equation from baseline serum creatinine^32^. CKD was defined as eGFR < 60 mL/min/1.73 m^2^ for 3 months or more.

### Spirometry

Spirometry was performed in individuals aged ≥ 40 years, except those for whom accurate results could not be obtained, such as those with history of laparotomy, ophthalmic surgery, open heart surgery, stroke, myocardial infarction, or cardiac arrest within the past 3 months; aortic aneurysm, retinal detachment, pneumothorax, urinary incontinence, or pulmonary tuberculosis; hemoptysis within the past 1 month; chest or abdominal pain; pain in the mouth or face when biting the mouthpiece; systolic blood pressure > 200 mmHg; diastolic blood pressure > 140 mmHg; dementia; reduced consciousness. Spirometry was performed by four technicians, each of whom underwent two education sessions on lung function tests and quality control. Trained technicians measured the FEV_1_, FVC and FEV_1_/FVC ratio using a dry rolling-seal spirometer (model 2130; Sensor Medics, Yorba Linda, CA, USA) and the American Thoracic Society/European Respiratory Society criteria for the standardization of lung function tests^33^. All of the spirometry values were described as prebronchodilator results^34^. Normal predictive values were derived taking into account age, sex, height, and ethnicity in large population studies of healthy subjects^35^. Analyses were performed only on data that met the following criteria: (i) 2 acceptable spirometry curves showing correct test initiation and expiration for at least 6 s and (ii) a greatest difference between two measurements of FEV_1_ or FVC of < 150 mL. The spirometry results were classified into three groups: normal and restrictive and obstructive lung function impairment. Participants with FEV_1_/FVC ≥ 0.7 and FVC ≥ 80% of the normal predicted value were considered normal. A restrictive pattern was defined as FEV_1_/FVC ≥ 0.7 and FVC < 80% predicted. An obstructive pattern was defined as FEV_1_/FVC < 0.7^36,37^.

### Clinical outcomes

The primary outcome was newly diagnosed non-valvular AF, either one diagnosis (ICD-10 code of I48.0-I48.4, I48.9) during hospitalization or more than two diagnoses in the outpatient clinics, until censoring or death^38^. A washout period of 1 year was set to ensure AF was newly diagnosed. To evaluate newly diagnosed AF, we used data derived from a 2007 cohort of the NHIS with clinical follow-up for the primary outcome through December 31, 2018.

### Statistical analysis

Summary statistics are expressed as the means and standard deviations for continuous variables and as numbers and percentages for categorical variables. Continuous variables were compared using Student’s t-test or analysis of variance, as appropriate. Categorical variables were compared using the chi-square test. Multiple comparisons were assessed using the Bonferonni correction. The IR of AF was calculated by dividing the number of AF cases by the sum of the follow-up duration and is presented as the rate per 1000 person-years. Participants were followed until the first diagnosis of AF, censoring by death, or December 31, 2018. Clinical outcomes were determined using the Kaplan–Meier method and compared using the log-rank test. Cox-proportional-hazard models were performed to analyze the impact of lung function on AF risk. The hazard ratio (HR) and 95% confidence interval (CI) were also calculated. A *p* value < 0.05 was considered statistically significant. To account for potentially confounding clinical covariates and to adjust the established risk factors for AF, multivariable Cox regression models were adjusted for age and sex (model 1); age, sex, BMI, household income, education, exercise, alcohol consumption, smoking, hypertension, DM, and dyslipidemia (model 2); and the variables in model 2 plus white blood cell count, eGFR, stroke, IHD, and asthma (model 3). Time-scale of the models was time on study. Statisticians confirmed the proportional hazard assumption through statistical tests based on Schoenfeld residuals and graphical diagnosis through log–log plots. In Fig. 1, *p* for trend was calculated through cox proportional regression analysis by considering the decile as a continuous variable. We also conducted subgroup analyses stratified by the confounding factors for the sensitivity analysis. The potential effect of modifications by the subgroups was evaluated using stratified analysis and interaction testing with a likelihood ratio test. All statistical analyses were performed using SAS version 9.4 (SAS Institute, Cary, NC, USA).

## Abbreviations

AF: Atrial fibrillation
DM: Diabetes mellitus
CKD: Chronic kidney disease
COPD: Chronic obstructive pulmonary disease
FEV_1_: Forced expiratory volume in 1 s
FVC: Forced vital capacity
IHD: Ischemic heart disease
KNHANES: Korean National Health and Nutrition Examination Survey
NHIS: National Health Insurance Service
BMI: Body mass index
WC: Waist circumference
ICD: International classification of disease
eGFR: Estimated glomerular filtration rate
HR: Hazard ratio
CI: Confidence interval
ARIC: Atherosclerosis risk in communities
MESA: Multi-ethnic study of atherosclerosis study

## References

[1] Benjamin EJ (2019). Heart disease and stroke statistics-2019 update: a report from the American heart association. Circulation.

[2] Hindricks G (2021). 2020 ESC Guidelines for the diagnosis and management of atrial fibrillation developed in collaboration with the European Association for Cardio-Thoracic Surgery (EACTS). Eur. Heart J..

[3] Krijthe BP (2013). Projections on the number of individuals with atrial fibrillation in the European Union, from 2000 to 2060. Eur. Heart J..

[4] Ganga HV, Nair SU, Puppala VK, Miller WL (2013). Risk of new-onset atrial fibrillation in elderly patients with the overlap syndrome: a retrospective cohort study. J. Geriatric Cardiol. JGC.

[5] Tattersall MC (2020). Persistent asthma is associated with increased risk for incident atrial fibrillation in the MESA. Circul. Arrhyth. Electrophysiol..

[6] Simons SO (2021). Chronic obstructive pulmonary disease and atrial fibrillation: an interdisciplinary perspective. Eur. Heart J..

[7] Sin DD, Wu L, Man SF (2005). The relationship between reduced lung function and cardiovascular mortality: a population-based study and a systematic review of the literature. Chest.

[8] Agarwal SK (2012). Airflow obstruction, lung function, and risk of incident heart failure: the Atherosclerosis Risk in Communities (ARIC) study. Eur. J. Heart Fail..

[9] Truelsen T, Prescott E, Lange P, Schnohr P, Boysen G (2001). Lung function and risk of fatal and non-fatal stroke. The Copenhagen City Heart Study. Int. J. Epidemiol..

[10] Johnson, L. S., Juhlin, T., Engström, G. & Nilsson, P. M. Reduced forced expiratory volume is associated with increased incidence of atrial fibrillation: the Malmo Preventive Project. *Europace : Eur. Pacing Arrhyth. Card. Electrophysiol. J. Work. Groups Card. Pacing Arrhyth. Card. Cell. Electrophysiol. Eur. Soc. Cardiol.* **16**, 182–188 (2014). 10.1093/europace/eut255 23960091

[11] Buch P, Friberg J, Scharling H, Lange P, Prescott E (2003). Reduced lung function and risk of atrial fibrillation in the Copenhagen City Heart Study. Eur. Respir. J..

[12] Li J (2014). Airflow obstruction, lung function, and incidence of atrial fibrillation: the Atherosclerosis Risk in Communities (ARIC) study. Circulation.

[13] Chahal H (2015). Ability of reduced lung function to predict development of atrial fibrillation in persons aged 45 to 84 Years (from the Multi-Ethnic Study of Atherosclerosis-Lung Study). Am. J. Cardiol..

[14] Kim BS (2019). The relationship between decreased pulmonary function and atrial fibrillation in general population: findings from Ansung-Ansan cohort of the Korean Genome and Epidemiology Study. J. Cardiol..

[15] Rodriguez, C. J. *et al.* Atrial fibrillation incidence and risk factors in relation to race-ethnicity and the population attributable fraction of atrial fibrillation risk factors: the Multi-Ethnic Study of Atherosclerosis. *Ann. Epidemiol.* **25**, 71–76, 76.e71 (2015). 10.1016/j.annepidem.2014.11.024 PMC4559265 25523897

[16] Elia, D. *et al.* Pulmonary hypertension and chronic lung disease: where are we headed? *Eur. Resp. Rev. Off. J. Eur. Resp. Soc.* **28** (2019). 10.1183/16000617.0065-2019 PMC9488824 31636088

[17] Walters TE (2014). Acute atrial stretch results in conduction slowing and complex signals at the pulmonary vein to left atrial junction: insights into the mechanism of pulmonary vein arrhythmogenesis. Circul. Arrhyth. Electrophysiol..

[18] Linz D (2018). Associations of obstructive sleep apnea with atrial fibrillation and continuous positive airway pressure treatment: a review. JAMA Cardiol..

[19] Rienstra M (2012). White blood cell count and risk of incident atrial fibrillation (from the Framingham Heart Study). Am. J. Cardiol..

[20] Fogarty AW, Jones S, Britton JR, Lewis SA, McKeever TM (2007). Systemic inflammation and decline in lung function in a general population: a prospective study. Thorax.

[21] Linz D (2019). Role of autonomic nervous system in atrial fibrillation. Int. J. Cardiol..

[22] Lahousse L, Verhamme KM, Stricker BH, Brusselle GG (2016). Cardiac effects of current treatments of chronic obstructive pulmonary disease. Lancet Resp. Med..

[23] Huerta, C., Lanes, S. F. & García Rodríguez, L. A. Respiratory medications and the risk of cardiac arrhythmias. *Epidemiology (Cambridge, Mass.)* **16**, 360–366 (2005). 10.1097/01.ede.0000158743.90664.a7 15824553

[24] Kim, Y. The Korea National Health and Nutrition Examination Survey (KNHANES): current status and challenges. *Epidemiol. Health* **36**, e2014002 (2014). 10.4178/epih/e2014002 PMC4017741 24839580

[25] Kweon S (2014). Data resource profile: the Korea National Health and Nutrition Examination Survey (KNHANES). Int. J. Epidemiol..

[26] Lee YH, Han K, Ko SH, Ko KS, Lee KU (2016). Data analytic process of a nationwide population-based study using national health information database established by national health insurance service. Diab. Metabol. J..

[27] Seo MH (2019). 2018 Korean society for the study of obesity guideline for the management of obesity in Korea. J. Obes. Metabol. Synd..

[28] Seo MH (2018). Prevalence of obesity and incidence of obesity-related comorbidities in Koreans Based on National Health Insurance Service Health Checkup Data 2006–2015. J. Obes. Metabol. Synd..

[29] Park S (2019). Dose-response relationship between cigarette smoking and risk of ulcerative colitis: a nationwide population-based study. J. Gastroenterol..

[30] Choi SI (2017). Relationship between regional body fat distribution and diabetes mellitus: 2008 to 2010 Korean National Health and Nutrition Examination Surveys. Diab. Metabol. J..

[31] Park, S. E. *et al.* Dose-dependent effect of smoking on risk of diabetes remains after smoking cessation: a nationwide population-based cohort study in Korea. *Diab. Metabol. J.* (2021). 10.4093/dmj.2020.0061 PMC8369207 33662197

[32] Matsuo, S. *et al.* Revised equations for estimated GFR from serum creatinine in Japan. *Am. J. Kidney Dis. Off. J. Natl. Kidney Found.* **53**, 982–992 (2009). 10.1053/j.ajkd.2008.12.034 19339088

[33] Miller MR (2005). Standardisation of spirometry. Eur. Respir. J..

[34] Kim SW (2016). The relationship between the number of natural teeth and airflow obstruction: a cross-sectional study using data from the Korean National Health and Nutrition Examination Survey. Int. J. Chronic Obstr. Pulm. Dis..

[35] Ranu H, Wilde M, Madden B (2011). Pulmonary function tests. Ulster Med. J..

[36] Park HJ (2015). Comorbidities in obstructive lung disease in Korea: data from the fourth and fifth Korean National Health and Nutrition Examination Survey. Int. J. Chronic Obst. Pulm. Dis..

[37] Godfrey MS, Jankowich MD (2016). The vital capacity is vital: epidemiology and clinical significance of the restrictive spirometry pattern. Chest.

[38] Kang SH (2016). Underweight is a risk factor for atrial fibrillation: A nationwide population-based study. Int. J. Cardiol..

